# Formation of Polyethylene Glycol Particles Using a Low-Temperature Supercritical Assisted Atomization Process

**DOI:** 10.3390/molecules24122235

**Published:** 2019-06-14

**Authors:** Hsien-Tsung Wu, Hong-Ming Tsai, Tsung-Hsuan Li

**Affiliations:** Department of Chemical Engineering, Ming Chi University of Technology 84 Gungjuan Rd., Taishan Dist., New Taipei City 24301, Taiwan; m05138212@mail2.mcut.edu.tw (H.-M.T.); lance19950803@gmail.com (T.-H.L.)

**Keywords:** low-temperature supercritical assisted atomization, polyethylene glycol

## Abstract

Polyethylene glycol (PEG) particles were prepared using low-temperature supercritical assisted atomization (LTSAA) with carbon dioxide as the spraying medium or the co-solute and acetone as the solvent. The effects of several key factors on the particle size were investigated. These factors included the concentration of the PEG solution, precipitator temperature, saturator temperature, ratio of the volumetric flow rate of carbon dioxide to the PEG solution, and the molecular weight of PEG. Spherical and non-aggregated PEG particles, with a mean size of 1.7–3.2 µm, were obtained in this study. The optimal conditions to produce fine particles were found to be a low concentration of the PEG solution, a low precipitator temperature, and low molecular weight of the PEG. The phase behavior of the solution mixture in the saturator presented a qualitative relationship. At the optimized volumetric flow rate ratios, the composition of CO_2_ in the feed streams was near the bubble points of the saturator temperatures. X-ray and differential scanning calorimetry analyses indicated that LTSAA-treated PEG had a reduced degree of crystallinity, which could be modulated via the precipitator temperature. PEG microparticles prepared by a LTSAA process would be promising carriers for drug-controlled formulations of PEG-drug composite particles.

## 1. Introduction

Polyethylene glycol (PEG) is a hydrophilic polymer manufactured by polymerization of ethylene oxide (EO) and is commonly used in the cosmetic, food, and pharmaceutical industries. PEG exhibits outstanding biocompatibility and toxicological safety. The World Health Organization (WHO) has set the acceptable maximum daily intake of PEG in foodstuffs as 10 mg/kg body weight [1]. In pharmaceutical applications, PEG has been used as laxatives, ointments, fillings for gelatin capsules, solubilizers, tablet excipients, and carriers to improve the dissolution rate of drug. Such enhanced dissolution properties were achieved by the micronization of the drugs, transformation of the drug into a crystalline or amorphous form, the formation of a solid dispersion, and the increase of the wettability of the drug through the use of a water-soluble carrier in composite particles [2,3,4,5]. In addition, hydrophilic PEG can mask the surface of hydrophobic materials and prevent recognition by the immune system, resulting in prolonged system circulation and bypass of the RES (reticuloendothelial system) [6,7].

The conventional processes for the fabrication of PEG solids include drum flaking, jet milling, spray drying [8], and spray freezing [9]. Supercritical carbon dioxide (scCO_2_) is a benign working medium and plays versatile roles in particle formation technologies, including RESS (rapid expansion of supercritical solutions), SAS (supercritical antisolvent), PGSS (particles from gas saturated solution), and SAA (supercritical assisted atomization). The RESS process is limited by the solubility of the polymer in scCO_2_ and the SAS process is not suitable for use in aqueous systems, due to the low solubility of water in scCO_2_. The yield of the PGSS process is greater, but larger PEG particles are produced [10,11]. The SAA process can be applied in both aqueous and organic solvent systems. However, solvents with lower boiling points should be utilized in the SAA process for micronization in the low-melting point of PEG (*T_m_* ≈ 335 K). Adami et al. [12] proposed a new configuration of SAA, operated at reduced pressure and temperature, to micronize the thermo-sensitive compounds poly L-lactide (PLLA) and bovine serum albumin (BSA). The same group [13] used this new SAA process to produce PEG microparticles and investigated the process parameters, including the molecular weight of PEG, the precipitator temperature, the concentration of the PEG solution, and the gas to liquid ratio (GLR). The aim of the present study is to integrally investigate the influence of the low-temperature supercritical assisted atomization (LTSAA) process parameters and the phase behavior on the size and morphology of the PEG particle and the properties of the resulting PEG powder.

## 2. Results and Discussion

**Figure 1:**
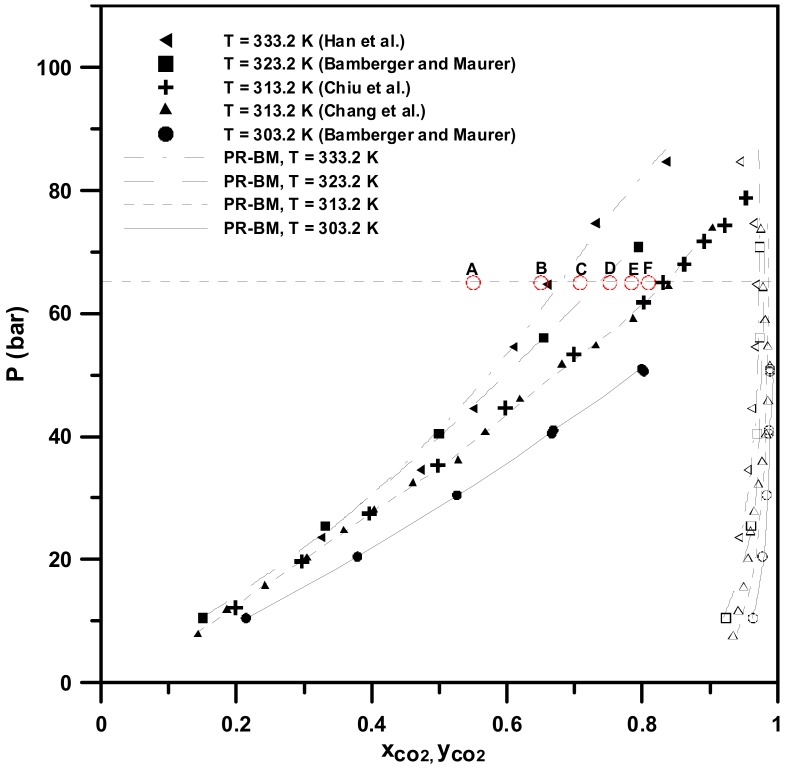
VLE phase diagram of the mixture of carbon dioxide + acetone. Points A, B, C, D, E, and F are referred to *R* values of 0.8, 1.2, 1.6, 2.0, 2.4, and 2.8, respectively.

The SAA involved two atomization steps, namely, pneumatic atomization and decompressive atomization. The efficiency of decompressive atomization depends on the content of the CO_2_ solubilized in the feed liquid solution in the saturator [14]. Therefore, the CO_2_ content in the feed liquid can be manipulated by adjusting the flow ratio of CO_2_ to the liquid solution, and is limited by the vapor-liquid equilibrium (VLE) phase envelopes. This hypothesis is verified by Reverchon and Antonacci [15] and our previous studies of PMMA, PMMA-co-BMA, and PMMA-co-EA particles produced via the SAA process. The composition of CO_2_ in the feed streams at the optimized flow ratio is close to the bubble point of the saturator temperature [16,17,18]. Thus, phase behavior is a crucial factor in selecting the proper conditions in the SAA process. The VLE phase boundaries of carbon dioxide + acetone were obtained using the Peng–Robinson Equation with the Boston–Mathias mixing rules [19] and are presented in Figure 1. The binary interaction parameters (*k_ij_* and *l_ij_*) are a function of temperature and have been used to fit experimental data at different temperatures, from 303.2 K to 333.2 K in literature [20,21,22,23]. The critical properties and acentric factors for the pure components and the binary interaction parameters used are presented in Table 1 and Table 2. The temperature range of the phase boundaries were included in the saturator temperature range in this study. These results could be used to qualitatively describe the relationship between the experimental conditions and the properties of the resulting PEG particles, as well as to select the appropriate conditions for the LTSAA procedure.

Mixing rule, as follows:

(1) $$a_{M}=\sum_{i}\sum_{j}x_{i}x_{j}\sqrt{a_{j}a_{j}}\left(1-k_{\mathrm{ij}}\right);\;k_{\mathrm{ij}}=k_{\mathrm{ij}}^{\left(1\right)}+k_{\mathrm{ij}}^{\left(2\right)}T+\frac{k_{\mathrm{ij}}^{\left(3\right)}}{T},$$

(2) $$b_{M}=\sum_{i}\sum_{j}x_{i}x_{j}\frac{b_{i}+b_{j}}{2}\left(1-l_{\mathrm{ij}}\right);\;l_{\mathrm{ij}}=l_{\mathrm{ij}}^{\left(1\right)}+l_{\mathrm{ij}}^{\left(2\right)}T+\frac{l_{\mathrm{ij}}^{\left(3\right)}}{T}.$$

### 2.1. Operation Parameters for LTSAA

The preliminary experiments were performed using conventional SAA with precipitation at atmospheric pressure [16], using a precipitation temperature of 333.2 K and a flow rate of 1.0 Nm^3^/h of heated N_2_. These experiments were unsuccessful, as a PEG film was obtained in the precipitator. This was attributed to the fact that the temperature used to evaporate the solvent during precipitation was too high for the low-melting point PEG, and thus induced particle coalescence. In subsequent experiments, the vacuum system was used to reduce the precipitator pressures and, correspondingly, lower the evaporation temperatures in the precipitator, i.e., the LTSAA process was implemented. In the preliminary LTSAA process, a precipitator pressure of 0.5 bar was achieved by the vacuum system, and a saturator temperature of 323.2 K, a solution flow rate of 3 mL/min, and a 1.0 Nm^3^/h flow rate of unheated N_2_ were used. The precipitator temperature (*T_p_*) could be decreased to 278 K, due to the endotherm of the evaporation heat of the solvent acetone and the Joule–Thomson effect induced by the expansion of CO_2_ downstream from the nozzle. Even though the precipitator temperature (*T_p_*) was lower than the boiling point of acetone at the precipitator pressure (*T_b_* = 308 K at 0.5 bar), PEG particles were successfully produced. This might be attributed to quick drying in the highly volatile acetone and the low solubility of PEG in low-temperature acetone, even if minor acetone condensation occurred in the precipitator chamber.

The precipitation experiments were conducted at precipitator temperatures (*T_p_*) of 273–293 K, a polymer solution concentrations (*C*) range of 10–50 mg/mL, a saturator temperature (*T_s_*) range of 313–333 K, and volumetric flow rate ratios of carbon dioxide to the polymer solution (*R* = *F_CO2_*/*F_l_*) of 0.8–2.8. The conditions of the flow of carbon dioxide were 276 K and 6.5 MPa. This state of CO_2_ was used to determine the density of CO_2_ and to calculate the mole fraction composition of the feed mixture in the saturator. Table 3 lists the experimental conditions and results of the PEG particles produced by the LTSAA process. In addition, to achieve a higher precipitator temperature under the fixed saturator temperature, CO_2_ flow rate, and PEG solution flow rate, the N_2_ temperature was set to 473 K and 373 K (Table 3, runs #1, 2). An unheated N_2_ flow was used in subsequent experiments.

### 2.2. Effects of the Precipitator Temperature and Concentration of the PEG Solution on the PEG Particle Size 

**Figure 2:**
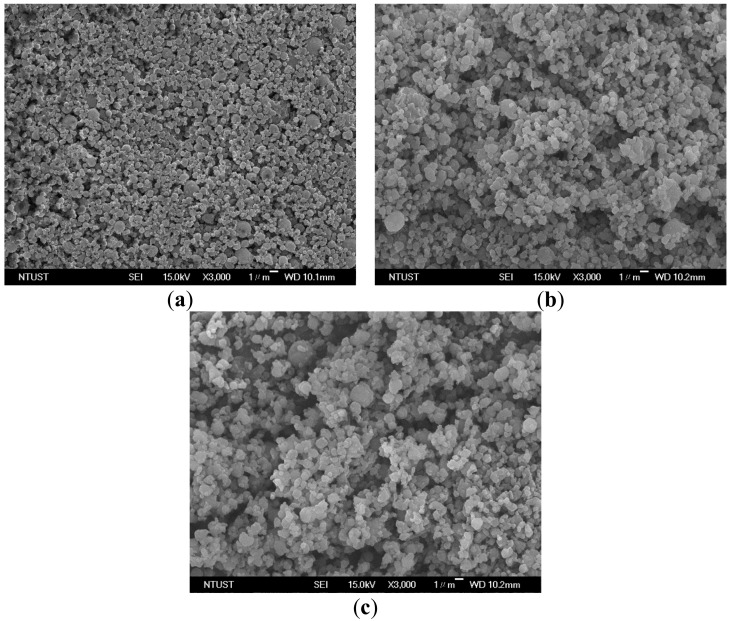
FESEM images of PEG particles produced by LTSAA process at different precipitator temperatures (*T_P_*): (**a**) *T_P_* = 278.2 K. (**b**) *T_P_* = 283.2 K. (**c**) *T_P_* = 293.2 K.

**Figure 3:**
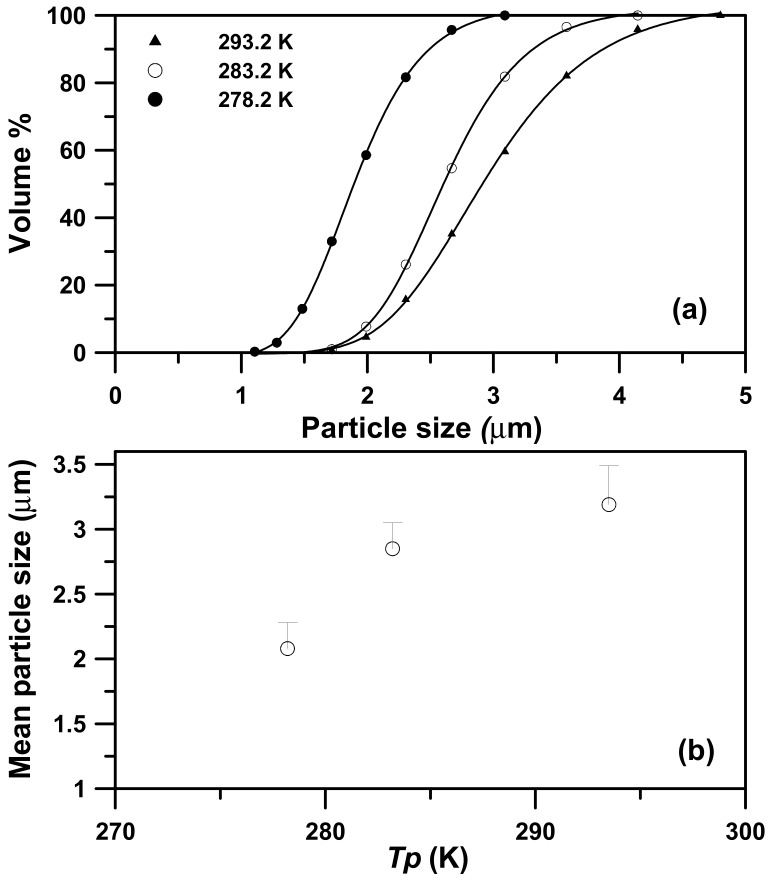
(**a**) The particle size distributions of the sample produced by the LTSAA process at different precipitator temperatures (*T_P_*), from 278.2 K to 293.2 K. (**b**) Mass-weighted mean particle size (◯) varying with precipitator temperature (*T_P_*) at *C* = 10 mg/mL, *T_S_* = 323.2 K, *R* = 2, and *MW* = 4000.

The precipitator temperature (*T_p_*) determines whether the micronization is successful for low-melting-point PEG. This study used LTSAA and the highly volatile solvent acetone to achieve the production of PEG microparticles within the precipitator temperature range of 278 K to 293 K. The effect of the precipitator temperature on the particle sizes was examined using the following fixed conditions: A concentration (*C*) of 10 mg/mL, a saturation temperature (*Ts*) of 323.2 K, and a volume flow ratio (*R*) of 2.0 (Table 3, runs #1 to #3). Figure 2 shows the field emission scanning electron microscopy (FESEM) images of the samples with spherical micrometric particles. The particle sizes distribution (PSD) (Figure 3a) and mean size of PEG particles (Figure 3b) increased with the precipitator temperature. Although a high temperature is conducive to rapid evaporation, it will cause the particles to soften and aggregate into larger particles. Similar results have been reported for other relevant SAA processes [13,24].

**Figure 4:**
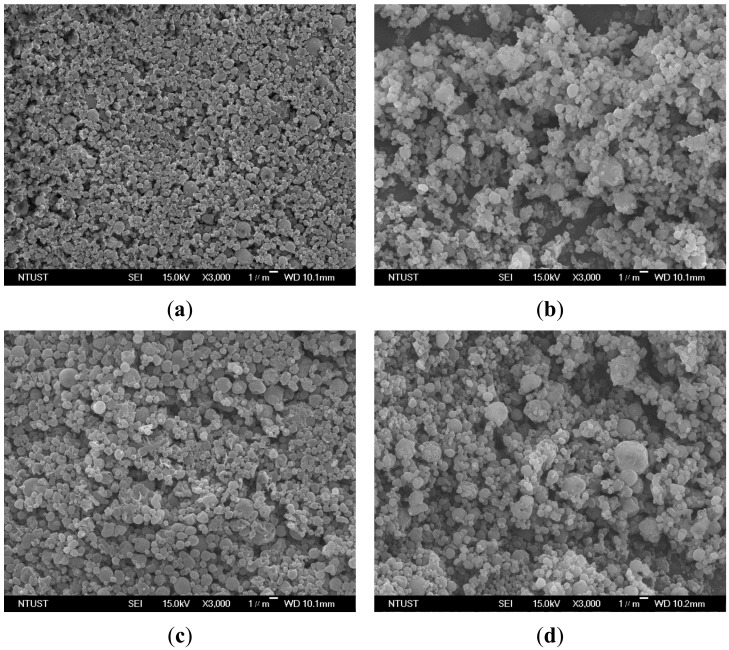
FESEM images of PEG particles produced by the LTSAA process at different concentrations of the PEG solution (*C*): (**a**) *C* = 10 mg/mL; (**b**) *C* = 20 mg/mL; (**c**) *C* = 30 mg/mL; (**d**) *C* = 50 mg/mL.

**Figure 5:**
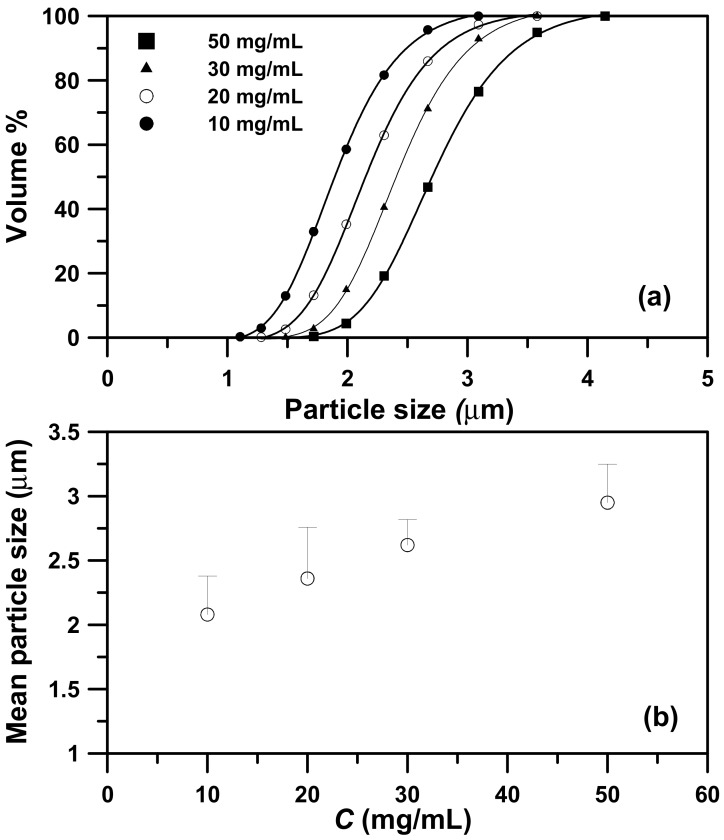
The particle size distributions of the sample produced by the LTSAA process at different concentrations of the PEG solution (*C*) from 10 mg/mL to 50 mg/mL. (**b**) Mass-weighted mean particle size (◯) varying with the concentration of the PEG solution (*C*) at *T_S_* = 323.2 K, *R* = 2, and *MW* = 4000.

The effect of the concentration of the PEG solution on the particle sizes was examined in the concentration (*C*) range of 10–50 mg/mL, at a saturator temperature (*Ts*) of 323.2 K, and a volume flow ratio (*R*) of 2.0 (Table 3, runs #3 to #6). Figure 4 shows the FESEM images of the samples with spherical micrometric particles. The PSD (Figure 5a) and mean size of the PEG particles (Figure 5b) increased with the concentration of the PEG solution, thus indicating that the high viscosity of the high concentration PEG solution resulted in larger liquid droplets and increased PEG particle sizes. Similar results have been reported, mostly for relevant SAA processes [13,14,15,16,17,18,25,26,27,28,29,30,31,32]. 

### 2.3. Effects of the Phase Behavior and Molecular Weight of PEG on the PEG Particle Size 

**Figure 6:**
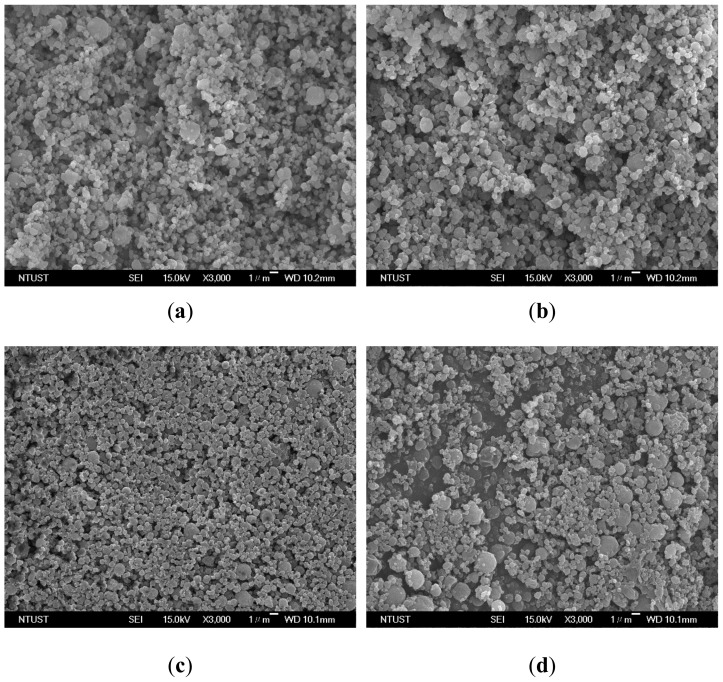
FESEM images of PEG particles produced by the LTSAA process at different volume flow ratios (*R*): (**a**) *R* = 0.8; (**b**) *R* = 1.5; (**c**) *R* = 2; and (**d**) *R* = 2.8.

**Figure 7:**
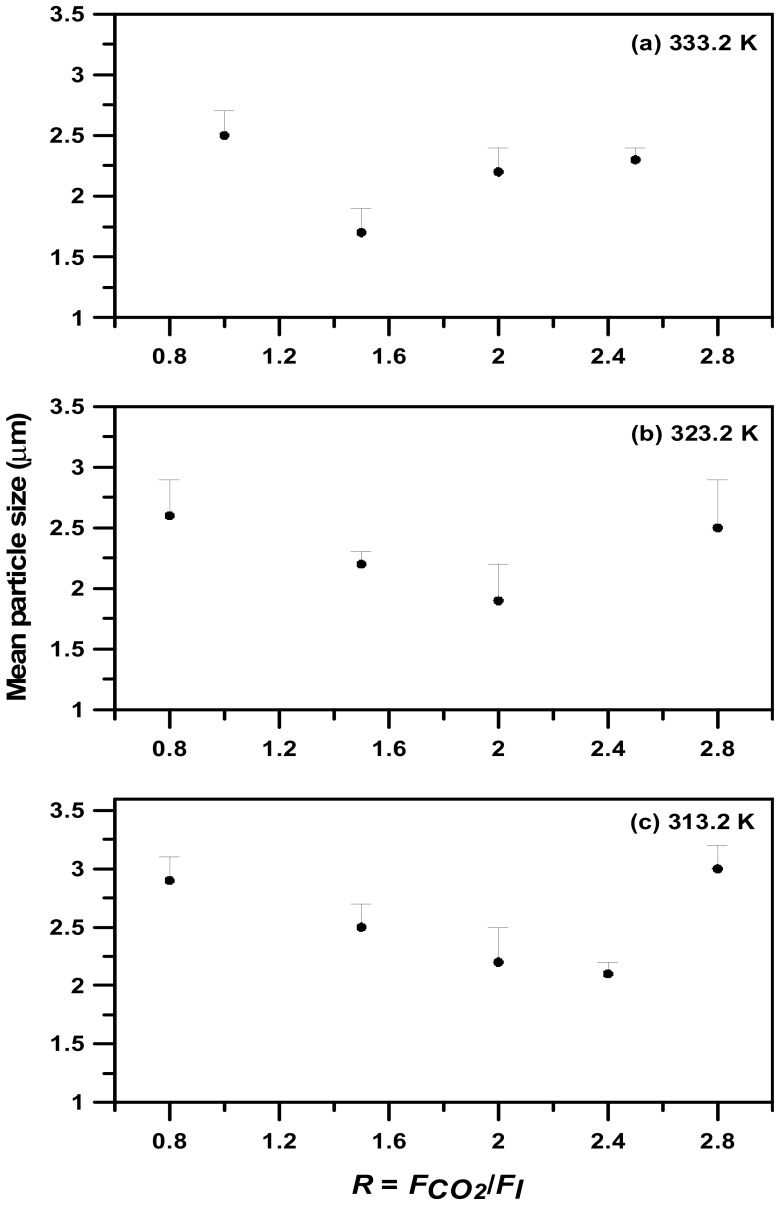
Mass-weighted mean particle size (●) varying with volume flow ratio (*R*) at *C* = 10 mg/mL and saturator temperatures: (**a**) *T_S_* = 333.2 K, (**b**) *T_S_* = 323.2 K, (**c**) *T_S_* = 313.2 K.

As described in Section 2, the composition of CO_2_ in the feed liquid can be manipulated by altering the flow ratio of CO_2_ to the liquid solution in the standard SAA process, and is limited by the VLE phase envelopes. However, the effect of phase behavior on the PEG particle size in the LTSAA process has not yet been investigated. The mean sizes of the PEG particles produced at different flow ratios (*R*) and saturator temperatures (*T_S_*) are presented in Table 3 (runs #3, #7 to #18). Figure 6 presents the FESEM images of samples with different flow ratios. Increasing *R* values favored smaller particles, but the optimal *R* value of 2.0 (Figure 6c) occurred at a saturator temperature of 323 K. The same results are also observed in Figure 7. The different saturator temperatures exhibited the following optimal *R* values: *R* = 1.5 at *T_S_* = 333.2 K; *R* = 2.0 at *T_S_* = 323.2 K; and *R* = 2.4 at *T_S_* = 313.2 K. 

Based on the VLE phase boundaries for different temperatures (Figure 1), the composition of CO_2_ in the saturated compressed-liquids (bubble point) was approximately *R* = 1.2–1.6 (333.2 K), *R* = 2.0 (333.2 K), and *R* = ~2.8 (313.2 K), respectively. These results were consistent with the optimized *R* values shown in Figure 7 at 333.2 K and 323.2 K. When the flow ratios were further increased, the operating conditions fell into the vapor-liquid coexistence region. The concentration of the PEG solution may have risen by acetone, soluble in CO_2_-rich vapor, producing the larger particles. The optimal *R* value was slightly less than that of the 313.2 K bubble point at *R* = 2.8, which was attributed to the anti-solvent effect induced by the fact that the mole fraction of CO_2_ in the saturated compressed-liquids was greater than 0.80. Based on these results and on previous studies of polymer micronization via the SAA process using acetone as the solvent [15,16,17,18], the importance of phase behavior in the LTSAA process was once again verified. 

**Figure 8:**
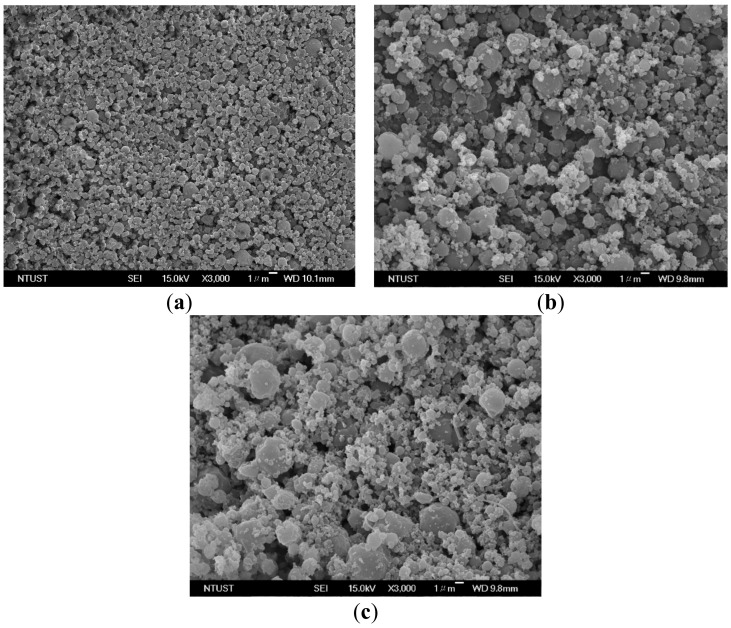
FESEM images of PEG particles produced by the LTSAA process at different molecular weights of PEG (*MW*): (**a**) *MW* = 4000, (**b**) *MW* = 6000, (**c**) *MW* = 10,000.

**Figure 9:**
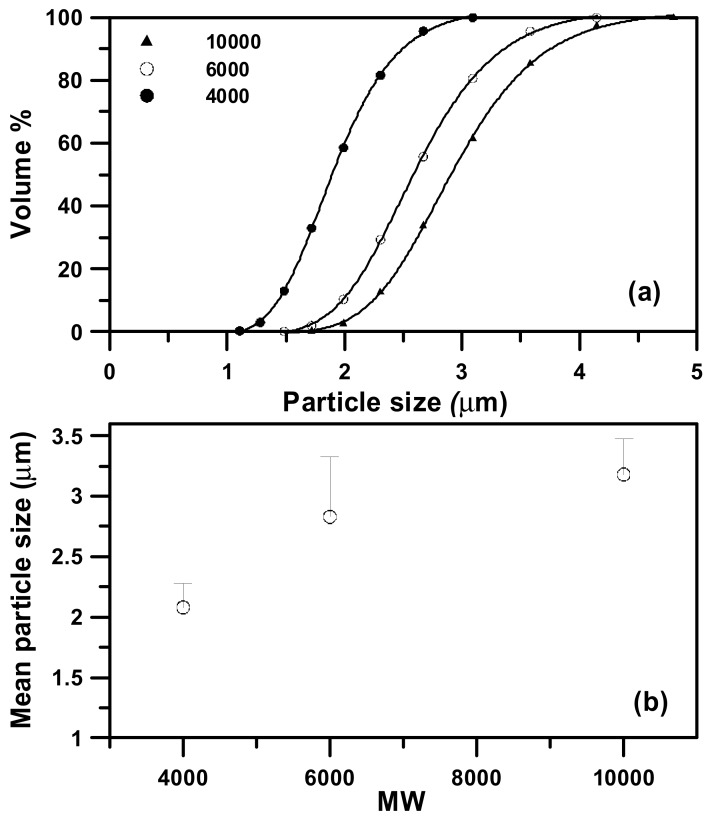
(**a**) The particle size distributions of the sample produced by the LTSAA process at different molecular weights of PEG (*MW*) from 4000 to 10000. (**b**) Mass-weighted mean particle size (◯) varying with molecular weight of PEG (*MW*) at *C* = 10 mg/mL, *T_S_* = 323.2 K, and *R* = 2.

The effect of the molecular weight (MW) of PEG on the particle size was also investigated in runs #3, #19 and #20. Figure 8 and Figure 9 present the FESEM images and PSD of samples with different MW. The mean sizes of PEG particles increase with the MW of PEG. This was attributed to the higher viscosity of a higher MW in the PEG solution. The same results were also presented in Liparoti et al. [13]. 

### 2.4. Solid Characterization 

**Figure 10:**
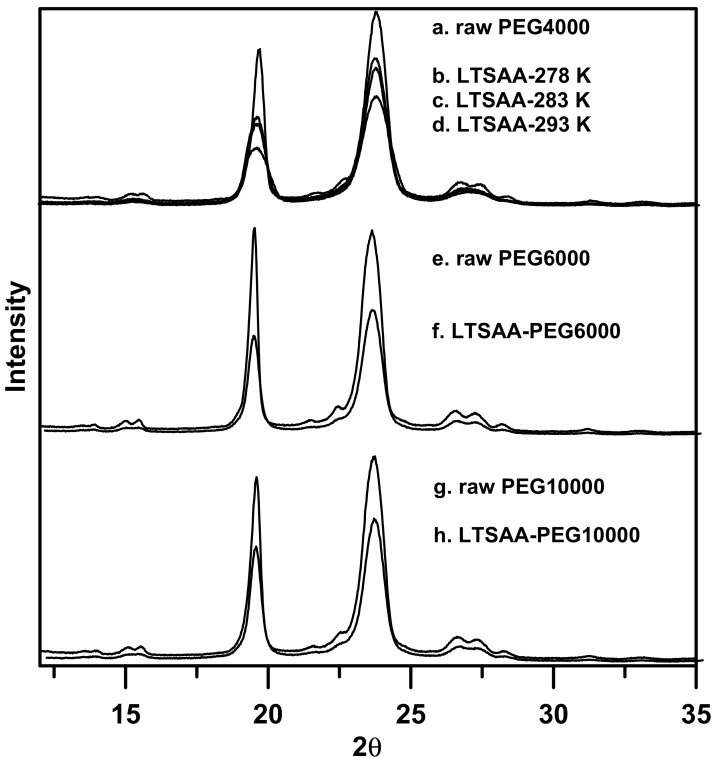
The XRD analyses of the PEG particles produced using LTSAA.

**Figure 11:**
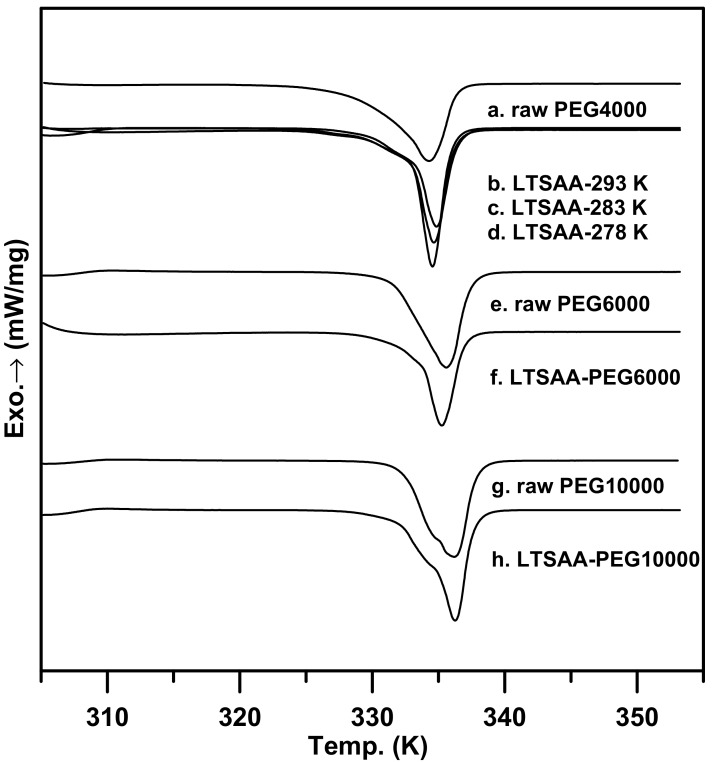
The DSC analyses of the PEG particles produced using LTSAA.

Figure 10 and Figure 11 show the X-ray diffraction (XRD) and differential scanning calorimetry (DSC) analyses of the as-received and LTSAA-processed PEG particles, respectively. The XRD results showed a reduction in the intensity of the characteristic peaks (19.2°, 23.4°) of all the LTSAA-PEG particles, compared to those of the as-receive PEG, which in turn suggested the presence of amorphous polymer and/or metastable folded forms in the LTSAA-PEG powder samples. Similar decreased crystallinity has been observed, mainly in SAA-related polymer particles, e.g., PMMA, PLLA, chitosan, and IMC-CH composite [3,12,15,31]. However, the presence of crystalline particles produced by the SAA process was still observed in some materials, e.g., mannitol and PEG, and was attributed to the crystallization rate being higher than the evaporation rate [13,32]. This hypothesis was demonstrated by the PEG particles prepared at different precipitator temperatures (Figure 10b–d). The magnitude of the XRD peak, 23.4°, was further reduced as the precipitator temperature was increased (23% to 42%). Based on the DSC analyses (Figure 11), the fusion heat of the raw PEG-4000 was 178.6 ± 0.5 J/g, while those of the LTSAA samples treated at precipitator temperatures of 278.2 K, 283.2 K, and 293.2 K (Figure 11d–b) decreased to 177.0 ± 0.4, 170.0 ± 0.6, and 155.0 ± 0.3 J/g, respectively. The LTSAA-treated samples with lower fusion heats exhibited reduced crystallinity at higher precipitator temperatures. 

For the LTSAA-treated samples of higher MW PEG particles (PEG6000 and PEG10000), the magnitude of the XRD peak at 23.4° reduced by 38% and 30%, respectively. A higher reduction in the magnitude of the XRD peak corresponded to higher reduction in the fusion heat of LTSAA-PEG6000 (141.2 ± 1.2 J/g), as compared with that of raw PEG6000 (182.2 ± 0.6 J/g). High MW PEG10000 exhibited lesser reduction in the magnitude of the XRD peak—the fusion heat of LTSAA-PEG10000 was174.8 ± 0.7 J/g, while that of raw PEG10000 was 178.2 ± 0.7 J/g. This could be attributed to the lower segmental mobility and more convenient geometrical alignment of high MW PEG [33]. The decrease in that magnitude of the XRD peaks and the fusion heats confirmed the reduction in the crystallinity of the LTSAA-treated PEG particles. Similar results were also presented for PEG particles prepared by spray drying from 95% ethanol [34]. Additionally, DSC analyses demonstrated that the melting points of PEG decreased with the molecular weight of PEG [35,36], and that the precipitator temperature and PEG particle size had a minor impact on the melting points of PEG. 

## 3. Materials and Methods 

### 3.1. Materials

Poly ethylene glycol (PEG, average MW = 4, 6, and 10 kg/mol) was purchased from Aldrich, USA. Acetone (99.9% purity, HPLC grade) was purchased from Tedia, USA. Carbon dioxide (99.9% purity) and nitrogen (99.9% purity) were purchased from Yung-Ping Gas Co., Taiwan. These chemicals were used without further purification.

### 3.2. Production of PEG Particles

**Figure 12:**
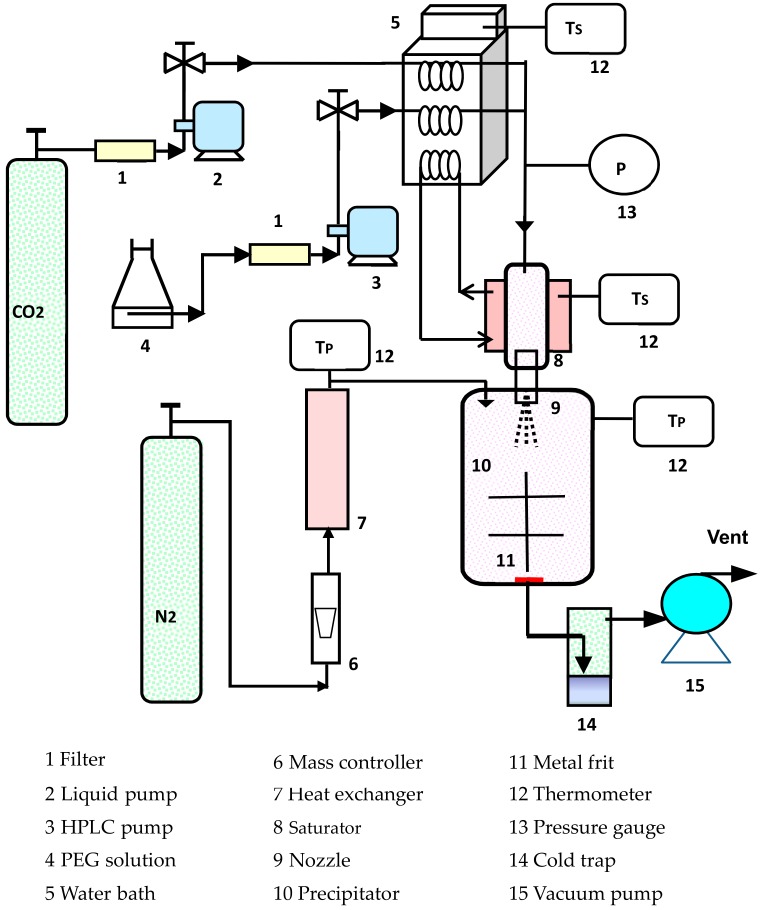
Apparatus of low-temperature supercritical assisted atomization (LTSAA).

Figure 12 shows a schematic diagram of the LTSAA apparatus installed in the present study. The apparatus consisted of a saturator (8), precipitator (10), cold trap (14), and three feeding lines. A high-pressure vessel (Model: 7973, 24 cm^3^, Applied Separations, Allentown, PA, USA) served as the saturator and was loaded with protruding stainless-steel packing that provides a large contact area between the PEG solution and the CO_2_. The solution in the saturator was sprayed through the nozzle (9) (internal diameter, 130 μm) into the precipitator. The saturator was located in a thermostatic circulating water bath (5), which was regulated to within ± 0.1 K. A metal frit (11) mounted at the bottom of the precipitator outlet retains the PEG particles. Downstream from the precipitator, a cold trap (14) (CT10/−50 °C, Firstek, Taipei, Taiwan) and vacuum pump (15) (G-100D, ULVAC, Chigasaki, Kanagawa, Japan) were used to recover the solvent and gas at reduced pressure. The three feeding lines contained the PEG solution (4), CO_2_, and N_2_. Two high-pressure liquid pumps were used to deliver CO_2_ (2) (NP-KX-540, Nihon Seimitsu Kagaku Co., Hon-cho, Kawaguchi, Japan) and the PEG solution (3) (PU-1580, JASCO, Hachioji, Tokyo, Japan). The N_2_ flow from the cylinder was controlled by a mass controller (6) (251-FKASBYAA, Porter, Hatfield, PA, USA) and heated in an electric heat exchanger (7) (series 93, Watlow, St. Louis, MO, USA), and was sent to the precipitator to facilitate the evaporation of the liquid droplets.

The experimental procedure is described here briefly. The CO_2_ flow rate (*F_CO2_*) and saturator temperature (*T_S_*) were preset. The saturator pressure was set at 6.5 ± 0.2 MPa. After a steady state was achieved, the PEG solution was introduced into the saturator at a flow rate of 3 mL/min. The PEG solution containing the dissolved CO_2_ was sprayed through the injection nozzle to atomize the liquid as it entered the precipitator. When the atomized solution came into contact with the heated N_2_ (1.0 Nm^3^/h), the solvent evaporated from the droplets, resulting in the supersaturation of the PEG particles. Samples of the product were collected from the precipitator and observed using FESEM (model 6500, JEOL, Akishima, Tokyo, Japan). 

The PSD and the mean size (the arithmetic mean particle sizes, *d_no_*, and the mass-weighted mean sizes, *d_4,3_*) of the PEG particle were determined using a dynamic light scattering (DLS) particle analyzer (Zatasizer Nano ZS90, Malvern, UK). Before analysis, the PEG particles were suspended in 278 K acetone and sonicated for 1 min. All precipitation experiments were performed in triplicate at minimum and used to calculate the mean values, with standard deviation, for each set of conditions. The average yield of the PEG particles using LTSAA was 80%. The weight loss was attributed to the adherence of some fraction of the PEG microparticles to the walls of the precipitator and inside the pores of the filter. 

### 3.3. Solid-State Characterization

The X-ray diffraction (XRD) patterns of the powder products were recorded using an X’Pert Pro X-ray powder diffractometer (PANalytical, Almelo, Netherlands). The scanning region of the diffraction angle (2θ) ranged from 10° to 50° at a scan rate of 0.02°/s. Thermograms of the PEG samples were obtained using low a temperature differential scanning calorimeter (LT-DSC, Netzsch 204 Fl Phoenix, Wittelsbacherstraße, Selb, Germany). Samples of 3–5 mg were placed in aluminum pans and the pans were sealed and heated from 298.2 K to 353.2 K at a rate of 5 K/min in N_2_ atmosphere. 

## 4. Conclusions

In this study, spherical and non-aggregated PEG particles were prepared using the LTSAA process, with CO_2_ as the spraying medium or the co-solute and acetone as the solvent. The experimental results showed that the mean size of the PEG particles decreased with the concentration of the PEG solution, precipitator temperature, and molecular weight of PEG. At the optimized volumetric flow rate ratios, the composition of CO_2_ in the feed streams was near the bubble points of the saturator temperatures. X-ray and DSC analyses indicated that LTSAA-treated PEG had a reduced degree of crystallinity, which could be decreased further by increasing the precipitator temperature. The different morphologies of PEG microparticles prepared by the LTSAA process would be promising carriers, and investigation of the dissolution rate of drug-controlled formulations of PEG-drug composite particles is in progress.

## Figures and Tables

**Table 1:** Critical properties and acentric factors for pure components.

Components	*T_C_* (K)	*P_C_* (bar)	ω
carbon dioxide	304.1	73.8	0.239
acetone	508.1	47.0	0.304

**Table 2:** Interaction parameters for carbon dioxide (1) + acetone (2) system.

$k_{\mathrm{ij}}$		$l_{\mathrm{ij}}$	
$k_{\mathrm{ij}}^{\left(1\right)}$	36.356	$l_{\mathrm{ij}}^{\left(1\right)}$	20.235
$k_{\mathrm{ij}}^{\left(2\right)}$	−0.05798	$l_{\mathrm{ij}}^{\left(2\right)}$	−0.03273
$k_{\mathrm{ij}}^{\left(3\right)}$	−5700.2	$l_{\mathrm{ij}}^{\left(3\right)}$	−3131.5

**Table 3:** Experimental conditions and results of PEG particles.

Run	*T_P_*	*C*	*T_s_*	*R*	*d_no_* *^a^*	*d_4,3_^b^*	*PDI*
	K	mg/mL	K	*F_CO2_/F_l_*	μm	μm	*d_4,3_/d_no_*
1 ^c^	293	10	323	2.0	2.9 ± 0.3	3.2 ± 0.3	1.1
2 ^d^	283	10	323	2.0	2.5 ± 0.2	2.9 ± 0.2	1.2
3	278	10	323	2.0	1.7 ± 0.2	2.0 ± 0.3	1.1
4	278	20	323	2.0	2.0 ± 0.3	2.3 ± 0.4	1.4
5	278	30	323	2.0	1.9 ± 0.2	2.5 ± 0.2	1.3
6	278	50	323	2.0	2.6 ± 0.2	2.9 ± 0.3	1.1
7	278	10	323	0.8	1.7 ± 0.1	2.6 ± 0.3	1.5
8	278	10	323	1.5	1.6 ± 0.2	2.2 ± 0.1	1.4
9	278	10	323	2.8	1.7 ± 0.1	2.5 ± 0.4	1.5
10	273	10	313	0.8	2.3 ± 0.2	2.9 ± 0.2	1.3
11	273	10	313	1.5	2.2 ± 0.2	2.5 ± 0.2	1.1
12	273	10	313	2.0	2.0 ± 0.3	2.2 ± 0.3	1.1
13	273	10	313	2.4	1.9 ± 0.4	2.1 ± 0.1	1.1
14	273	10	313	2.8	2.6 ± 0.4	3.0 ± 0.2	1.2
15	285	10	333	1.0	2.0 ± 0.3	2.5 ± 0.2	1.3
16	285	10	333	1.5	1.5 ± 0.2	1.7 ± 0.2	1.1
17	285	10	333	2.0	1.6 ± 0.5	2.2 ± 0.2	1.3
18	285	10	333	2.5	1.6 ± 0.5	2.3 ± 0.1	1.4
19 ^e^	278	10	323	2.0	2.5 ± 0.3	2.9 ± 0.5	1.2
20 ^f^	278	10	323	2.0	2.6 ± 0.3	3.2 ± 0.3	1.2

^a^*d_no_*_._ = $\frac{\sum_{i=0}^{N}\mathrm{Di}}{N}$, arithmetic mean size. ^b^ *d_4,3_* = $\left(\frac{\sum_{i=0}^{N}{\mathrm{Di}}^{4}}{\sum_{i=0}^{N}{\mathrm{Di}}^{3}}\right)$, mass-weighted mean size. *^c^ T_N2_ = 473 K*. ^d^ *T_N2_* = 373 K. ^e^ MW = 6 kg/mole (molecular weight of PEG) ^f^ MW = 10 kg/mole (molecular weight of PEG).
